# Microcystin-degrading bacteria reduce bioaccumulation in *Fragaria vulgaris* and enhance fruit yield and quality

**DOI:** 10.1007/s11356-024-34568-0

**Published:** 2024-08-28

**Authors:** Mohammed Haida, Fatima El Khalloufi, Yasser Essadki, Diogo A. M. Alexandrino, Richard Mugani, Abdessamad Hejjaj, Alexandre Campos, Vitor Vasconcelos, Maria F. Carvalho, Leticia Díez-Quijada, Ana M. Cameán, Brahim Oudra

**Affiliations:** 1 Water, Biodiversity and Climate Change Laboratory, Faculty of Sciences Semlalia, Cadi Ayyad University, Marrakesh, Morocco; 2 Natural Resources Engineering and Environmental Impacts Team, Multidisciplinary Research and Innovation Laboratory, Polydisciplinary Faculty of Khouribga, Sultan Moulay Slimane University of Beni Mellal, B.P: 145, 25000 Khouribga, Morocco; 3 CIIMAR, Interdisciplinary Centre of Marine and Environmental Research, Terminal de Cruzeiros Do Porto de Leixões, Av. General Norton de Matos, S/N, 4450-208 Porto, Portugal; 4 Department of Environmental Health, School of Health, P. Porto, Porto, Portugal; 5 Department of Biology, Faculty of Sciences, University of Porto, Rua Do Campo Alegre, 4169-007 Porto, Portugal; 6 National Center for Studies and Research On Water and Energy, Cadi Ayyad University, P.O Box: 511, 40000 Marrakech, Morocco; 7 School of Medicine and Biomedical Sciences (ICBAS), University of Porto, Rua de Jorge Viterbo Ferreira 228, 4050-313 Porto, Portugal; 8 Area of Toxicology, Faculty of Pharmacy, University of Sevilla, C/Profesor Gacia Gonzalez 2, 41012 Seville, Spain

**Keywords:** Microcystins, *Ensifer sp.*, *Shinella sp.*, *Stutzerimonas* sp., Biodegradation of MCs, Growth, Plant yield, Physicochemical quality attributes

## Abstract

In Morocco, red fruit production has thrived, primarily utilizing hydroponic methods to control crops, increase fruit yield and quality, and avoid soil-related problems. However, the irrigation of these expansive hydroponic farms relies heavily on water sourced from dams, many of which are contaminated with Microcystins (MCs). To address this contamination issue, ongoing research is focused on discovering effective and cost-efficient biological solutions for eliminating MCs. In this study, we isolate and identify bacterial strains capable of degrading MCs, evaluate the rate of degradation, and investigate how soil inoculated with these bacteria affects the accumulation of MCs in plant tissue. The partial 16S rRNA analyses of three bacterial sequences were conducted, identifying them through NCBI as follows: *Ensifer* sp. (B1) isolated from soil, *Shinella* sp. (B2) from a cyanobacterial bloom, and *Stutzerimonas* sp. (B3) from water. These bacteria exhibited the ability to degrade MCs, with approximately 34.75%, 73.75%, and 30.1% of the initial concentration (20 µg/L) being removed after a 6-day period for B1, B2, and B3, respectively. Moreover, strawberry plants were cultivated hydroponically in a greenhouse for a duration of 90 days. These plants were subjected to extracts of cyanobacteria containing 10 and 20 µg/L of Microcystins (MC), as well as water from an artificial lake contaminated with MC, both with and without the presence of isolated bacterial strains. Among these strains, *Shinella* sp. exhibited the highest efficacy in mitigating MC accumulation. Specifically, it resulted in a reduction of approximately 1.159 µg of MC per kilogram of root dry weight, leading to complete elimination in the leaves and fruits. The findings also indicated that the inoculation of perlite with the three MC-degrading bacterial strains significantly enhanced growth, photosynthetic pigments, yield, biochemical constituents, and quality attributes of strawberries (p ≤ 0.05). These promising outcomes suggest the potential of this approach for addressing the adverse impacts of crops irrigated with MC-contaminated water in future agricultural practices.

**Supplementary Information:**

The online version contains supplementary material available at 10.1007/s11356-024-34568-0.

## Introduction

Microcystins (MCs) are toxins produced by cyanobacteria during blooms in nutrient-rich freshwater environments (Huisman et al. 2018; Mutoti et al. 2024). These toxins are a significant global environmental concern due to their capacity to cause a range of water safety issues (Pflugmacher et al. 2001; Imanishi et al. 2005. MCs possess a seven-peptide cycle structure, which imparts them with chemical resilience against variations in pH, temperature, sunlight (Marrs 1996; Li et al. 2011; Rastogi et al. 2014).

The practice of irrigating agricultural land, utilizing soilless cultivation methods such as hydroponics, and employing water contaminated by MCs sourced from freshwater ecosystems experiencing cyanobacterial blooms, along with the application of cyanobacterial compost and bank filtration techniques, can result in the introduction of MCs into terrestrial ecosystems (Chen et al. 2012; Corbel et al. 2015; Machado et al. 2017). This can result in high concentrations of MCs, reaching levels of up to a few milligrams per kilogram (Chen et al. 2012). The hydrophilic nature of MCs in the soil facilitates their absorption by plants through their root systems, leading to their accumulation in consumable plant parts. This accumulation further promotes their multiplication within food chains, ultimately resulting in grave repercussions for the well-being of both humans and animals (Gutiérrez-Praena et al. 2014).

Exposure to MC can have diverse effects on human health. It triggers hepatotoxicity and inhibits protein phosphatases, leading to significant protein phosphorylation in liver cells. This biochemical disruption is associated with the development of liver and colorectal cancers, along with disturbances in various physiological processes (Dawson 1998). Furthermore, MCs exhibit diverse phytotoxic effects, including reduced seed germination, hindered plant growth and development, compromised photosynthetic performance, and disturbances in hormonal balance. These toxins also alter plant metabolisms, such as increased lipid peroxidation and oxidative stress, and decreased protein content. Consequently, the yield losses attributable to MC exposure can range from 50 to 82%, depending on the specific crop and the duration of exposure (Saqrane et al. 2008; El Khalloufi et al. 2011, 2012; Cao et al. 2018a; Gu and Liang 2020).

The persistence of MCs in natural waterways varies depending on several agents, this persistence ranging from 6 to 120 days (Chen et al. 2006; Li et al. 2011; Cao et al. 2018a). To mitigate the persistence of MCs in water and to combat their hepatotoxicity and their harmful effects on ecosystems and public health, much research has been carried out on methods for degrading MCs (Eleuterio and Batista 2010; Wu et al. 2011; Zhou et al. 2016). Biodegradation has attracted a lot of interest as an environmentally friendly and cost-effective approach to managing MC contamination, outperforming conventional treatment methods. Currently, it is emerging as the leading method for MC removal from water and soil sources due to its exceptional efficiency, moreover, this approach does not require continuous maintenance or chemicals that may produce undesirable by-products (Ho et al. 2012; Zhao et al. 2013; Li et al. 2014).

In the biodegradation process, various heterotrophic bacteria from different environments have demonstrated their ability to effectively degrade MCs. Notable examples include the genus *Sphingomonas*, as well as various species belonging to the genera *Acinetobacter*, *Arthrobacter*, *Bacillus*, *Novosphingobium*, and *Paucibacter* (Massey and Yang 2020). Moreover, studies have shown that the utilization of multiple bacterial strains, forming a bacterial community, has been effective in degrading MCs. Investigation into the bacterial metabolism involved in MC degradation has identified four key genes (*mlrA*, *B*, *C*, *D*), three intracellular hydrolytic enzymes (MlrA, B, C), and three resulting degradation products (linearized MC-LR, tetrapeptide, and Adda). Additionally, various research endeavors on *Sphingopyxis* have uncovered eight new intermediate products derived from different MC variants, illuminating the degradation pathway through Phenylacetic acid (PAA) metabolism. This pathway identifies CO_2_ as the ultimate degradation product, offering valuable insights into the hydrolysis process. (Massey and Yang 2020).

The specific objectives of this study were 1) to isolate and purify bacterial strains capable of degrading MCs from different media and to evaluate their degradation kinetics; 2) determine the efficacy of plant inoculation by these bacteria in reducing the bioaccumulation of MCs in a hydroponic culture.; 3) assess the impact of reduced bioaccumulation of MCs on the growth, physiological, biochemical, and nutritional properties of strawberry plants, as well as on fruit quality attributes. This study's significance lies in providing insights into the direct application of bacteria as a means to remove MCs from water, thereby mitigating the ecological risks associated with MCs poisoning and enhancing the quality of cultivated edible products, especially in hydroponic crops.

## Materials and methods

### Isolation and molecular identification of bacteria strain

For the isolation of bacteria, 50 mL of cyanobacterial bloom were added to 100 mL flasks and placed in a shaking incubator that was set to run for four hours at 140 rpm. This action was used to make it easier for the bacterial strains to be dislodged and separated from the cyanobacterial colonies. For soil sample, 10 g of soil from a farm irrigated with reservoir water contaminated by MCs were mixed with 90 ml of sterile physiological water and continuously agitated at 180 rpm. For water bacteria, 1000 ml of surface water from Lake Lalla Takerkoust was filtered using a 0.7 µm glass microfiber filter to effectively remove larger particles. For isolation, the mineral salt medium (MSM) composed of 1.6 g of K_2_HPO_4_, 0.4 g of MgSO_4_-7H_2_O, 0.5 g of NaCl, 20 mg of CaCl_2_, and 2.3 mg of FeCl_3_-6H_2_O per 1000 mL of water were utilized as the mineral salt medium (MSM). A crude extract of MC-LR was extracted and prepurified on a C18 cartridge, and added to the medium as the sole source of carbon and nitrogen. Subsequently, 100 μl from each sample was plated onto MSM-agar. These plates were then incubated at 28°C for a duration of 48 to 72 h. Purification of the strains was achieved through repeated inoculation of morphologically distinct colonies on the same MSM-MC-agar. Subsequently, pure strains were further grown on nutrient agar and Tryptic Soy Agar (TSA) media to check the purity and were stored in a 25% glycerol solution at -20°C (Shen et al. 2019).

MC-tolerant strains were phylogenetically identified by 16S rRNA gene sequencing. Genomic DNA was extracted from biomass of pure cultures grown on TSA using the E.Z.N.A.® Bacterial DNA Kit (Omega Bio-tek, Inc., Georgia, USA), according to the instructions of the manufacturer. Then, the DNA of each isolate was amplified by Polymerase Chain Reaction, as described previously by Alexandrino et al. (2018), and the resulting amplicons showing an appropriate size (ca. 1500 bp) were sent for Sanger sequencing at the Genomics i3S Scientific Platform (Porto, Portugal). Contig sequences of the 16S rRNA genes of each isolate were generated using the Geneious software (version 11.1.4) and compared to the 16S ribosomal RNA sequences database of the National Center for Biotechnology Information (NCBI) for taxonomic identification. The 16SrRNA gene sequences obtained for each isolate were deposited in GenBank® (NCBI, Maryland, USA) under the accession numbers OR753275-OR753277. The phylogenetic tree was generated using Maximum Likelihood and Tamura-Nei model (Tamura and Nei 1993). It included 15 nucleotide sequences with a total of 1673 positions, and MEGA11 (Tamura et al. 2021) was used for the analysis.

### Extraction of cyanotoxins

Freeze-dried *Microcystis aeruginosa* algal bloom biomass (250 mg) was obtained from the Lalla Takerkoust reservoir near Marrakech, Morocco in October 2010. The biomass was homogenized in water and sonicated in an ice bath for 3 min at a frequency of 42 kHz to lyse the cells and release the intracellular MCs. Following sonication, the resultant mixture was centrifuged at 10,000 g for 15 min to separate the cell debris from the liquid extract. The extraction process was done twice to achieve maximal recovery of MCs. A protein phosphatase type 2A inhibition test was used to measure the total concentration of MCs. The overall MC concentration in this experiment was 11.5 mg of MCs per gram of dry weight DW). Furthermore, evaluation of MC variations using High-Performance Liquid Chromatography (HPLC) revealed that MC-LR was the dominant MC variant, accounting for 95% of the total MCs present (El Khalloufi et al. 2013).

### Screening bacterial strains tolerant to microcystin

To assess the bacterial strains' tolerance to MC, they were cultivated in 250 mL of liquid nutrient agar containing two different concentrations of MCs (10 µg/L and 20 µg/L of MCs). These strains were then subjected to incubation in a shaking incubator at 150 rpm and 28°C for 6 days. Following the incubation period, the growth was assessed by measuring the optical density at a wavelength of 600 nm in samples taken at intervals of 0, 2, 4, and 6 days (Chen et al. 2010).

### Kinetics of microcystin biodegradation by bacterial strains

Using the extract obtained from the bloom of *Microcystis aeruginosa*, two concentrations of MCs, 10 µg/L and 20 µg/L were prepared. In 100 ml flasks, these quantities were combined with potassium phosphate buffer (pH 7.0, 50 mM). The flasks were placed on a shaker set to 150 rpm and kept at 30°C. A volume of 5 mL was sampled for each treatment at 0, 2, 4, and 6-day intervals. This procedure was repeated twice, and the average data was examined. To serve as a control, a reaction mixture lacking microorganisms was employed (Zhang et al. 2011). The 5 ml aliquots were centrifuged, and the filtrate was passed through C18 Solid Phase Extraction (SPE) cartridges, specifically Oasis HLB cartridges (3 mL, 3 cc) from Waters (catalog number WAT094226), using a Manifold vacuum system. These cartridges were activated by 5 mL of 99% methanol through them and then rinsing them with 5 mL of deionized water. Toxins are recovered after elution with 5 ml methanol/formic acid solution (95:5 v/v), then the fraction was evaporated to dryness using a rotary evaporator. The residues were recovered in 1 ml of deionized water and stored at -80°C until ELISA analysis.

The total MCs content was quantified using the Abraxis Microcystins-ADDA ELISA kit from Eurofins (Warminster, PA, USA). This ELISA kit is used for the detection of MCs and nodularins, with a detection limit of 0.1 µg/L of MCs. The ELISA assay was conducted in strict accordance with the provided kit instructions. An RT-2100 microplate reader, Version 2.0e (OPTIC ivymen® SYSTEM, Guangdong, China), was employed to measure the absorbance at 450 nm. The calibration curve was established using a certified MC-LR standard at various concentrations (0, 0.15, 0.4, 1, 2, and 5 µg/L). A control of MC-LR (0.75 µg/L) was employed, and the ELISA assay was valid when a close value (0.75 ± 0.185 µg/L) was obtained. MCs in samples were determined in duplicates, and they were expressed as µg/L.

### Inoculation and plant growth promotion experiment in hydroponic systems

Inocula of *Ensifer sp.* (B1*)*, *Shinella sp.* (B2), and *Stutzerimonas* sp. (B3) were generated by culturing strains in nutrient agar at 28°Cwith agitation at 140 rpm for 5 days. Bacterial cells were extracted and washed three times with sterile physiological water before being resuspended in a sufficient volume of sterile physiological water to achieve a final OD_600_ of 1 (about 10^9^ CFU/mL). Two concentrations of MCs (10 µg/L and 20 µg/L) were prepared from the extract of *Microcystis aeruginosa* bloom, these two concentrations are realistic, since the concentrations of microcystins in the dam studied do not exceed 40 µg/L as a maximum value. The strains prepared were used to inoculate the roots of strawberry plants. The plants were inoculated with 5 ml of bacteria 3 times, once a month near the roots. The purpose was to test a user-friendly method of inoculation that is easily applicable in large-scale agriculture units.

Three-leaf strawberry (*Fragaria Vulgaris*) plants were carefully selected and placed in two-liter pots containing perlite sterilized with bleach and rinsed well several times for hydroponics for 90 days in a greenhouse. All 32 pots, with 6 duplicates for each treatment: C: control group; C1: group of plants irrigated with 10 µg/L of MCs; C2: group of plants irrigated with 20 µg/L of MCs; CDL: group of plants irrigated with lake water contaminated with MCs; C1 µg/L + B1: group of plants irrigated with water containing 10 µg/L of MCs in the presence of strain B1; C2 µg/L + B1: group of plants irrigated with water containing 20 µg/L of MCs in the presence of strain B1; CDL µg/L + B1 dam water contaminated with MCs (4.12 µg/L) in the presence of strain B1; C1 µg/L + B2: group of plants irrigated with water containing 10 µg/L of MCs in the presence of strain B2; C2 µg/L + B2: group of plants irrigated with water containing 20 µg/L of MCs in the presence of strain B2; CDL µg/L + B2 dam water contaminated with MCs in the presence of strain B2; C1 µg/L + B3: group of plants irrigated with water containing 10 µg/L of MCs and with strain B3; C2 µg/L + B3: group of plants irrigated with water containing 20 µg/L of MCs and with strain B3; CDL + B3 dam water contaminated with MCs in the presence of strain B3. The three bacterial strains were inoculated, in the perlite sterilized pots, near the roots, Three MC treatments were tested, 10 µg/L, 20 µg/L and Lalla Takerkoust Lake water (4.12 µg/L of MCs). A control without bacterial inoculation irrigated with the same MCs treatments and also with uncontaminated water was done3 times a week. Each treatment was conducted in sex duplicates. The greenhouse experiment was conducted on a farm in the Lalla Takerkoust region of Marrakech between November 2021 and February 2022, under natural conditions of humidity, temperature, sunlight, and photoperiod. To eliminate any potential impact of the microsite, the pots were moved randomly every 7 days during the growing period.

### Plant harvesting, growth parameter assessment, and plant quality evaluation

The plants were carefully taken out of the pots on the last day of the exposure period (90 days), washed with distilled water, and divided into roots, leaves, and fruits. After the various morphometric parameters of the plant and fruit were determined.

### Determination of photosynthetic pigments

To extract chlorophylls and carotenoids from leaf samples, we followed the method outlined by Upadhyay and Pame 2019. We used 5 mL of 95.5% acetone to extract these compounds from 0.5 g of leaves. To quantify chlorophyll a, chlorophyll b, and carotenoids, we employed a UV–visible spectrophotometer (Cary 50 Scan, Australia) at wavelengths of 662 nm, 644 nm, and 470 nm, respectively. The concentrations of Chl a, Chl b, total chlorophyll, and carotenoids in the leaf tissue were determined and expressed in mg/g FW.

$$\text{Chl a}=\text{9,784 }{\text{DO}}_{662}-\text{0,99 }{\text{DO}}_{644}$$

$$\text{Chl b}=\text{21,42 }{\text{DO}}_{644}-\text{4,65 }{\text{DO}}_{662}$$

$$\text{Total chlorophyll}=\text{Chl a}+\text{Chl b}$$

$$\text{Caro}=1000\; {\text{DO}}_{470}-1.90\text{ Chl a}-63.14\text{ Chl b}/214$$

### Determination of MCs in plant tissues

To evaluate the accumulation of MCs in various parts of the strawberry plant, we used the method described by Corbel et al. 2016. Fresh strawberry tissues, including roots, leaves, and fruit, were pulverized into a powder using liquid nitrogen in a mortar. Following that, we homogenized 0.5 g of root tissue, 1 g of leaves, and 2 g of fruit in 2 mL, 7 mL, or 20 mL, respectively, of an aqueous methanol–water (75% v/v) mixture. The mixtures were centrifuged at 4 °C for 10 min at 4000 g, and the filtrate was passed through C18 Solid Phase Extraction (SPE) cartridges, specifically Oasis HLB cartridges (3 mL, 3 cc) from Waters (catalog number WAT094226), using a Manifold vacuum system. The activation of these cartridges was initiated by passing 5 mL of methanol (99%) through them, followed by a 5 mL rinse with deionized water. Toxins are recovered after elution with 5 ml methanol/formic acid solution (95:5 v/v), then the fraction was evaporated to dryness using a rotary evaporator. The residues were recovered in 1 ml of deionized water and stored at -80°C until ELISA analysis.

### Estimation of daily intake

$$\text{EDI}={\text{C}}_{\text{MC}}\text{ x }{\text{M}}_{\text{fruit}}/ {\text{M}}_{\text{body}}$$

Where C_MR_ is the concentration in fruit; M_fruit_ is the fruit mass consumed per day (33 g for adults and 16.5 g for children); M_body_ is the body mass (60 kg for adults and 25 kg for children) (Jia et al. 2018).

### Determination of total sugar content in fruit

To analyze the concentration of soluble sugars, 0.1 g of fruit was pulverized and mixed with 4 mL of 80% ethanol. The resulting homogenate was then subjected to centrifugation at 5000 g for 10 min. Next, 1 mL of the supernatant was combined with 1 mL of 5% (v/v) phenol solution and 5 mL of sulfuric acid. After a 5-min incubation period, the optical density was determined at 485 nm using a UV–visible spectrophotometer (Square 50 Scan, Australia). The measurement of soluble sugar content was conducted with a glucose solution reference curve (Dubois et al. 1956).

### Determination of total protein content in fruit

For extraction, 0.5 g of fresh fruit was powdered with liquid nitrogen in a cold mortar and homogenized in 5 mL of a solution containing 50 mM potassium phosphate buffer (pH 7.0), 5% (w/v) polyvinylpolypyrrolidone, and 0.1 mM ethylenediaminetetraacetic acid (EDTA). The extracts were then centrifuged at 4° C for 20 min at 12,500 g, and the supernatants were used to determine the total protein content (Latef and Chaoxing 2011). The Bradford technique (Bradford 1976) was used to determine total protein concentration. A 2 ml reaction mixture containing 1500 µl of 0.1 M phosphate buffer (pH 7), 100 µl of extract, and 400 µl of Bradford reagent was prepared. The optical density was measured at 595 nm after 4 min of incubation. Protein content was calculated as mg. g^−1^ DW using a standard curve produced using bovine serum albumin (BSA) standard solutions.

### Determination of vitamin C content in fruit

A titrimetric method was employed for the determination of ascorbic acid content (Pathy 2018). Initially, 0.5 g of fresh fruits were roughly ground in a mortar, and then they were treated with 3 mL of 2% HCl, allowing the mixture to stand for 10 min. After this, the vitamin extract was centrifuged at 5000 rpm for 10 min at 4 °C. The resulting extract was combined with 3 mL of distilled water in an Erlenmeyer flask, and three drops of 0.5% starch solution were introduced as an indicator. The mixture was subsequently titrated using 0.01 N iodine solution, and this titration process was performed in four replicates. The endpoint of the titration was determined when the first dark blue-black solution was observed.

### Determination of total phenolic compounds in fruit

To determine the content of these phenolic compounds, we followed the method initially outlined by Kähkönen et al. 1999 with slight adjustments. Approximately 0.2 g of *F. vulgaris* roots or leaves per treatment were homogenized in 95% methanol. The resultant homogenate underwent centrifugation at 30,000 g for 10 min. Subsequently, 1 mL portions of the extracts were combined with 1 mL of 1 N Folin–Ciocalteau reagent and 1 mL of 10% sodium carbonate, and this mixture was incubated for 1 h at 35 °C. The absorbance was measured at 530 nm, and the total phenolic content was expressed in mg of gallic acid equivalents per g of FW.

### Determination of inorganic ions in fruit

The concentration of Ca^2+^ and K^+^ ions was determined according to the procedure established by (Pequerul et al. 1993). Freeze-dried samples weighing 0.25 g were first calcined for 6 h at 550 °C in a muffle furnace. The mineral residues obtained were then dissolved in 3 ml of 6 N hydrochloric acid and evaporated at 250 °C in a fume hood. The particles were reconstituted in 3 mL of hot distilled water (at 100 °C) and filtered through Whatman paper (0.45 μm). Each sample was then adjusted to a final volume of 50 ml. Flame emission photometry was used to determine Ca^2+^ and K^+^ concentrations. Data reported in mg/L.P contents were determined using the sodium molybdate method (Olsen and Sommers 1982). The other minerals Fe^2+^, Cl^−^, and Zn were measured in the fruit using X-ray fluorescence spectrometry (XRF).

### Statistical analysis

Analysis of the various parameters was carried out in six replicates per treatment. Results are expressed as mean ± standard error (SE). Differences between treatments were assessed by one-way ANOVA, and means were compared by Tukey's HSD test. Significant differences at *p* < 0.05 are indicated by different letters. A two-factor analysis of variance (ANOVA) was performed to compare the interaction between treatments with MC concentrations and the same MC concentrations in the presence of MC biodegrading bacteria. Significant differences in two-way ANOVA were achieved at *p* < 0.05, ** *p* < 0.01, and *** *p* < 0.001 for the different factors. The ANOVA was performed using the statistical software SPSS version 22.0.

## Results and discussion

### Isolation and identification of MC-degrading bacteria

**Fig. 1:**
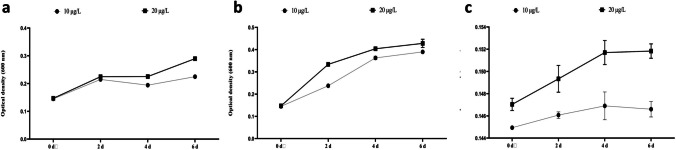
Growth curves for bacterial isolates B1 (**a**), B2 (**b**), and B3 (**c**) in the presence of MC at 10 and 20 µg/L in the nutrient medium

**Fig. 2:**
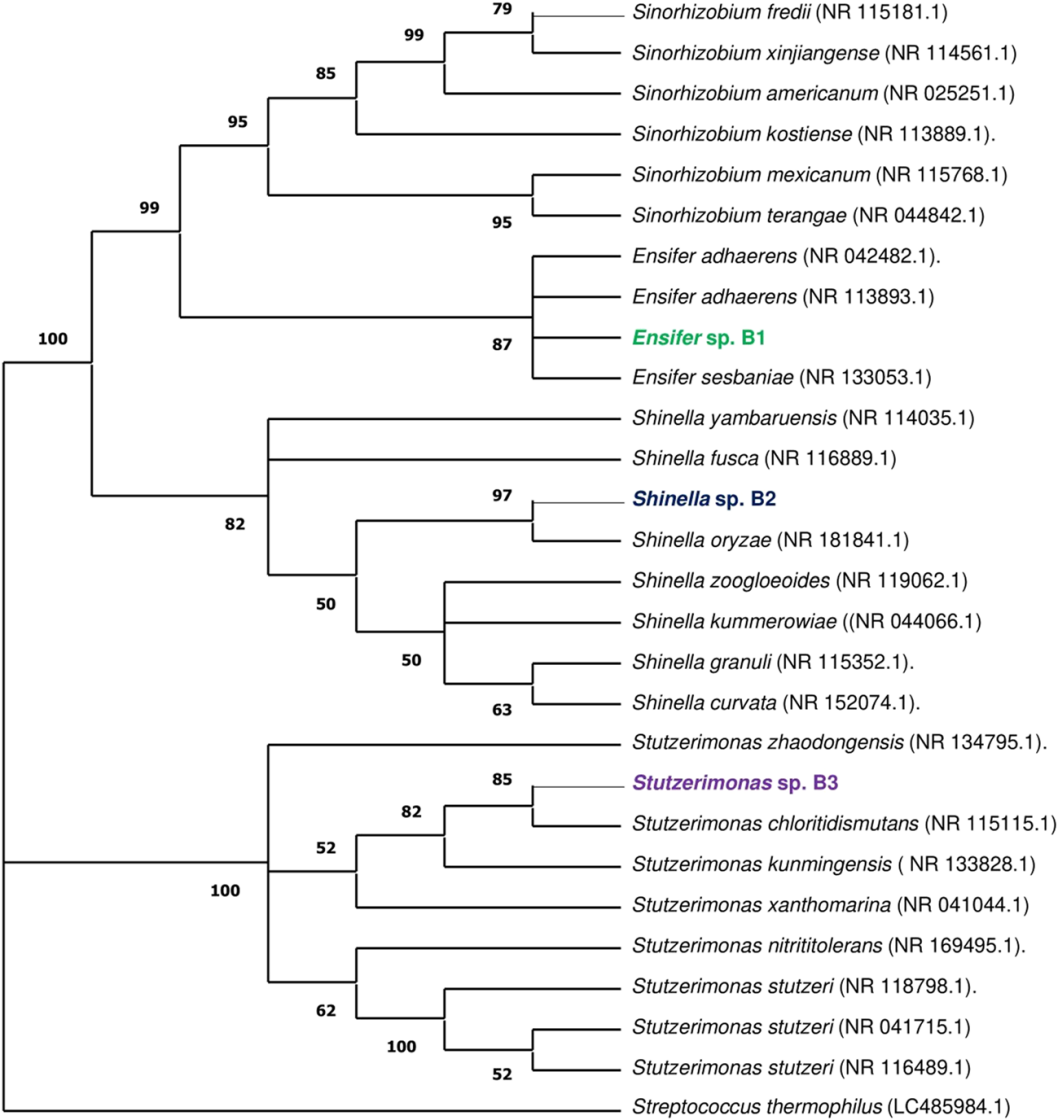
Maximum Likelihood Phylogenetic Tree of 16S rRNA Sequences using Tamura-Nei Model, depicting clustering patterns and evolutionary relationships of 28 sequences (1569 positions) in MEGA

During the isolation process, a total of 52 bacterial strains were obtained from the cyanobacterial bloom, 36 strains from the water, and 44 strains from the soil. Among all the isolated strains, three were selected for the study based on their growth performance on MSM-MC-agar. One strain with the best size and rapid growth was chosen from each matrix. They were cultivated at 28.5 °C in 250 ml flasks that were filled with liquid nutrient agar. These cultures were supplemented with 10 and 20 µg/L of MC for 6 days. The density measurements taken after 48 h indicated that two of the isolates, Bacteria 1 and Bacteria 2, exhibited rapid growth. Specifically, Bacteria 1 (B1), Bacteria 2 (B2), and Bacteria 3 (B3) achieved their maximum growth with optical density (OD) values of 0.28 nm, 0.42 nm, and 0.15 nm, respectively, in the medium containing 20 µg/L after the 6 days (Fig. 1). Strains B1, B2 and B3 were taxonomically classified as *Ensifer* sp., *Shinella* sp. and *Stutzerimonas* sp., respectively, based on 16S rRNA gene sequencing and analysis (Fig. 2).

**Fig. 3:**
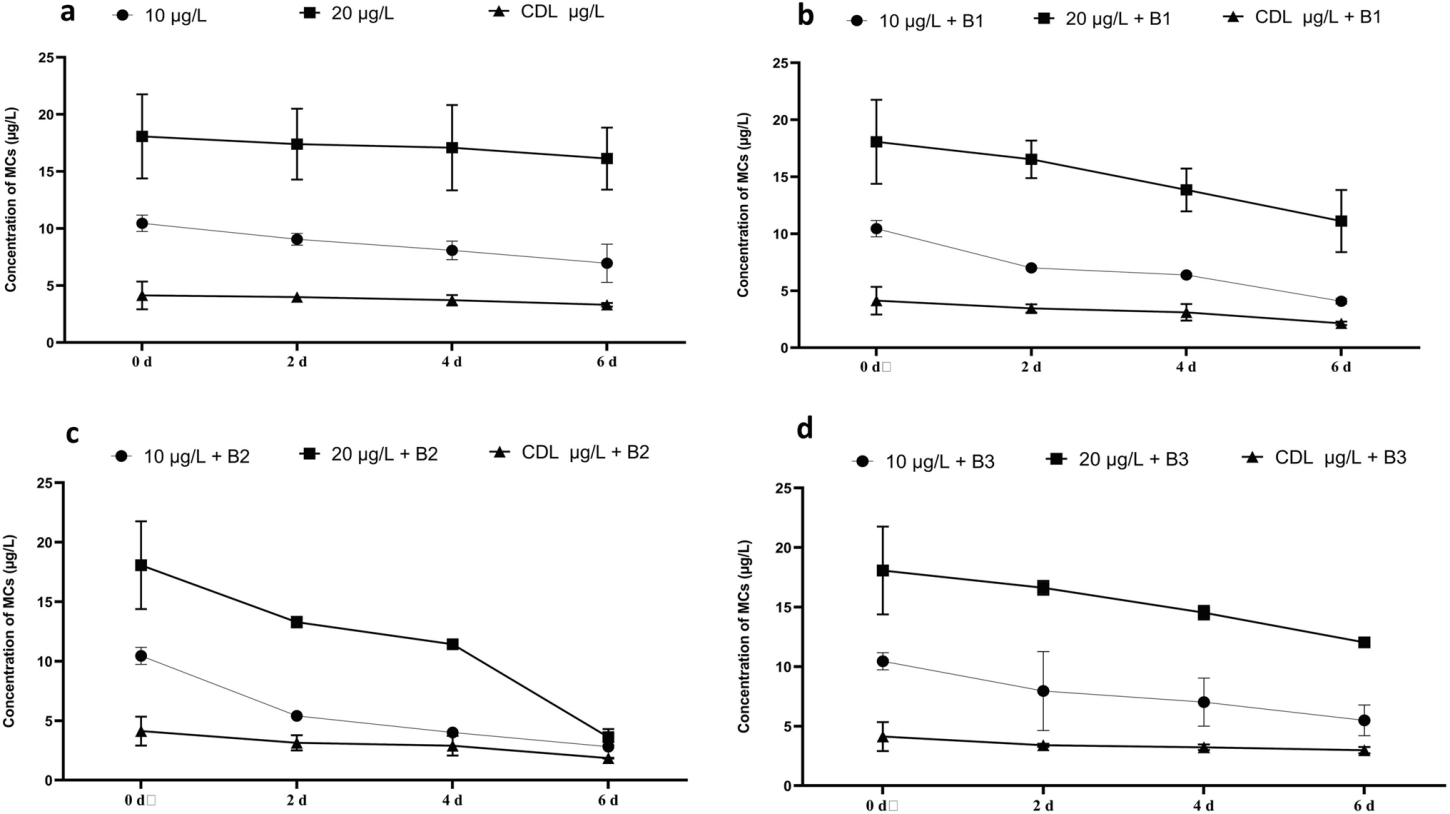
Decay kinetics of MC in the absence of MC-degrading bacteria (**a**), in the presence of B1 bacteria (**b**), in the presence of B2 bacteria (**c**), and in the presence of B3 bacteria (**d**)

In this investigation, we assessed the capacity of three distinct bacterial strains, *Ensifer sp.* (B1*)*, *Shinella sp.* (B2), and *Stutzerimonas* sp. (B3) (Fig. 3), to resist MC in a liquid medium. The intention behind this evaluation was to subsequently employ these bacterial strains in the process to attenuate the toxic effects of MCs in strawberry seedlings. Throughout this experiment, it was observed that bacterial growth in the MC-enriched medium followed an exponential pattern over six days. Interestingly, the presence of MCs had a discernible impact on the growth of B1, B2, and B3. It should be noted that the presence of MCs can normally inhibit or have adverse effects on bacterial growth (Lukhele and Msagati 2023). These results suggest its MC-tolerant bacteria may have the potential ability to degrade this molecule.

### Kinetics of biodegradation of microcystins

Figure 3 illustrates the kinetics of MC degradation in the presence and absence of MC-degrading bacteria. When MC-degrading bacteria were absent, the concentration of MC decreased over 6 days, going from 10.44 to 6.94 µg/L, 18.06 to 16.12 µg/L, and 4.12 to 3.30 µg/L in media containing 10, 20, and CDL µg/L of MC, respectively (Fig. 3a). In contrast, with the introduction of bacteria 1 into the medium, which initially had MC concentrations of 10.44, 18.06, and 4.12 µg/L, the concentrations decreased to 7, 16.52, and 3.44 µg/L after 2 days and further decreased to 4.06, 11.11, and 2.13 µg/L of MC after 6 days in media containing 10, 20, and CDL µg/L of MC, respectively (Fig. 3b). Bacteria 2 exhibited a more rapid MC degradation, averaging 7.63 µg/L in a medium containing 10 µg/L, 14.75 µg/L in a medium containing 20 µg/L, and 2.27 µg/L in the dam water (CDL) after 6 days (Fig. 3c). This rate of degradation was higher than that observed for bacterium 3, which had an average degradation of 4.96 µg/L in a medium containing 10 µg/L, 6.02 µg/L in a medium containing 20 µg/L of MC, and 1.14 µg/L in the dam water (CDL) after 6 days (Fig. 3d).

Preliminary results from the selection process demonstrated the ability of all three isolates to effectively reduce MC levels in the phosphate buffer solution. This reduction followed a consistent exponential pattern over a period ranging from 2 to 6 days, strongly suggesting that MCs degradation is the result of biological activity rather than natural processes. This result holds promise for mitigating the toxic impact of MCs for instance in plant production systems contaminated with MCs. Prior research has established that specific coexisting bacteria exhibit a notably efficient ability to break down MCs compared to natural factors like sunlight, heat, and substrate adsorption, as documented in previous studies (Harada and Tsuji 1998). The degradation process of MCs by bacteria comprises several key stages: an enzyme known as MlrA initiates the opening of the MC-LR ring at the Adda-Arg peptide bond, resulting in the formation of linearized MC-LR. Subsequently, the second enzyme, MlrB, further breaks down linearized MC-LR into a tetrapeptide. Finally, the enzyme MlrC hydrolyzes the tetrapeptide into its constituent smaller amino acids (Harada and Tsuji 1998). Notably, these degradation products, namely linearized MC-LR, the tetrapeptide, and Adda, were found to be essentially non-toxic. These results suggest that using MC-degrading bacteria is a highly effective method for detoxifying MCs (Imanishi et al. 2005).

### Bioaccumulation of MC in strawberry plant tissue

Table 1 shows the results from the assessment of MC quantity accumulated within various parts of strawberry plants (roots, leaves, and fruit) using ELISA. After irrigating the plants with MC-contaminated water for 90 days, in the absence of MC-degrading bacteria, the results indicate significant accumulation. Specifically, the roots showed an accumulation of around 9.12, 12.90, and 2.67 µg. Kg^−1^ FW when exposed to irrigation with 10, 20 μg/L, and with dam lake water contaminated with MCs, respectively. Similarly, the leaves displayed accumulations of 4.08, 7.266, and 1.74 µg. Kg^−1^ FW under the same respective MC concentrations, while the fruit exhibited accumulations of 3.02, 4.51, and 1.69 µg. Kg^−1^ FW. The treatment of plants exposed to MC by the B1 bacteria showed a significant reduction in the bioaccumulation of MC in the roots. The content increased from 9.12 to 3.30, and from 12.90 to 4.66 µg/L of MC, respectively. On the other hand, in plants treated with dam water (CDL µg/L) in the presence of the B1 bacteria, total elimination of MCs in the roots was noted. It's important to note that treatment with this bacterium did not induce bioaccumulation in the leaves and fruit, regardless of the MC concentration used. As well as total elimination of MCs was recorded after treatment with 10 and CDL µg/L in the presence of B2 bacteria in the roots. Treatment with 10, 20, and CDL µg/L in the presence of bacterium 3 (B3) showed a significant decrease in the concentration of MCs to 8.21, 11.06, and 1.84 µg. Kg^−1^ FW of MC in roots, respectively. In leaves, a decrease to 3.61 and 11.32 µg. Kg^−1^ of MC was observed after treatment with 10 and 20 µg/L + B3. MCs were not detected in fruit after treatment with CDL µg/L + B3. The bioaccumulation of MCs in edible crops has received increasing attention in recent years, as shown by various studies. In this study, MC accumulation in roots, leaves, and fruits was high after irrigation with a high concentration of toxin (20 µg/L), while the lowest accumulation was observed after irrigation with CDL µg/L in strawberry plants not treated with MCs-degrading bacteria. According to numerous studies, several crops accumulate a considerable quantity of MCs in the roots rather than in the shoots, such as maize (*Z. mays*), durum wheat (*T. durum*) (Saqrane et al. 2009), rice (*O. sativa*) (Liang and Wang 2015; Cao et al. 2018b; Liang et al. 2021) and legumes such as peas (*P. sativum*), lentils (*L. esculenta*) and *faba beans* (Saqrane et al. 2009; Redouane et al. 2021a, b). The highest quantities of MC were observed in the roots compared with the leave, since the roots are the first point of contact between the plant and the MCs. However, higher concentrations of MCs can be found in the stem and leaves and this has been well demonstrated in various plant species, especially in the case of spray irrigation (Cao et al. 2018a; Saqrane et al. 2009; Jiang et al. 2024). The bioaccumulation of MCs varies from one plant to another and depends on the concentration of MCs, the duration of exposure, and the type of MCs used (artificial or natural) (Haida et al. 2023).

**Table 1:** Bioaccumulation of MCs (μg MC.kg^−1^ FW) in the different tissues (roots, leaves, and fruit) of *Fragaria vulgaris* during a 90-day culture with exposure to 10, 20 μg/L, and CDL μg/L of MCs, and after treatment with three MC-degrading bacteria (*Ensifer* sp. (B1), *Shinella* sp. (B2), and *Stutzerimonas* sp. (B3))

Treatment		0 µg/L	10 µg/L	20 µg/L	CDL µg/L	10 µg/L + *Ensifer* sp*.*	20 µg/L + *Ensifer* sp.	CDL µg/L + *Ensifer* sp.	10 µg/L + *Shinela* sp.	20 µg/L + *Shinella* sp.	CDL µg/L + *Shinella* sp.	10 µg/L + *Stutzerimonas* sp.	20 µg/L + *Stutzerimonas* sp.	CDL µg/L + *Stutzerimonas* sp.
MCs in strawberry tissues (μg kg ^−1^)	**Roots**	nd^a^	9.124 ± 0.170^f^	12.907 ± 0.497^ g^	2,671 ± 0.173^d^	3.304 ± 0,254^d^	4.667 ± 0.125^e^	nd^a^	nd^a^	1.159 ± 0.033b^c^	nd^a^	0.918 ± 0.012^b^	1.846 ± 0.130^c^	0.839 ± 0.037^b^
	**Leaves**	nd^a^	4.080 ± 0.031^a^	7.266 ± 0.029^e^	1.747 ± 0.002^f^	nd^a^	nd^a^	nd^a^	nd^a^	nd^a^	nd^a^	0.471 ± 0.007^b^	1.582 ± 0.011^c^	nd^a^
	**Fruit**	nd^a^	3.026 ± 0.138^c^	4.518 ± 0.039^b^	1.696 ± 0.025^a^	nd^a^	nd^a^	nd^a^	nd^a^	nd^a^	nd^a^	nd^a^	nd^a^	nd^a^

Different letters indicate significant differences at *p* < 0.05

A decrease in MC bioaccumulation was evident with the presence of three bacterial strains, denoted as B1, B2, and B3. Bacteria 2 (B2) exhibited a notably high capacity to reduce MC bioaccumulation, followed by bacteria 1 (B1) and 3 (B3). These findings align with previous research, where *Rhizobium sp*. bacteria have been recognized for their MC-degrading capabilities (Levizou et al. 2020). Furthermore, another author reported the capacity of a strain of *Rhizobium selenitireducens* to degrade MCs, and the gene responsible for this biodegradation process has been meticulously identified (Do Carmo Bittencourt-Oliveira et al 2016). Similarly, the bioaccumulation pattern in faba bean shoots appeared distinct, with high levels observed in sterilized soil and lower levels in undisturbed soil. This suggests that in the absence of soil microflora, MCs bypass the biodegradation process, becoming more accessible to plants (Redouane et al. 2021a, b). Consequently, they accumulate in the stem, leaf, and fruits of the plants. To the best of our understanding, when MCs find their way into agricultural soils through irrigation water, their availability to plants hinges on several factors, including the specific type of toxin, soil characteristics, soil chemistry, and the microorganisms inhabiting the rhizosphere. Consequently, the interaction of MCs with soil particles and their decomposition by native flora play a vital role in diminishing their accumulation in plants, as documented in various studies (Miller and Fallowfield 2001; Edwards et al. 2008; Parmar and Sindhu 2013; Shen et al. 2019). Furthermore, it has been noted that in hydroponic cultivation, plants encounter a higher exposure to free MCs compared to plants grown in soil. Free MCs in irrigation water are prone to more rapid degradation, particularly when high concentrations of MC-degrading bacteria are present in the water (Nautiyal 1999; Shen et al. 2019; Redouane et al. 2021a, b). In our current research, we have sought to use microorganisms to reduce MCs in the hydroponic system and reduce the bioaccumulation of MCs. This biological approach is inspired by a phenomenon commonly observed in freshwater ecosystems. Our new degradation method holds promise for practical applications in controlling the release of MC following the lysis of toxic cyanobacteria.

### Estimated daily intake

The EDI values for both adults and children of the fruits of plants irrigated with 10, 20, and CDL µg/L exceed the tolerable daily intake (TDI) limit (0.04 µg Kg^−1^d^−1^) proposed by WHO (Table 2). the EDI values ​​arrive at 1.58, 2.36 and 0.84 after consumption of fruits irrigated with 10, 20 and CDL µg/L of MC, respectively by adults, and 1.99, 2.98 and 1.11 after consumption of fruits irrigated with 10, 20 and CDL µg/L µg/L of MC, respectively by children. The application of 3 bacteria effectively lowered the EDI values ​​below the TDI limit till none, whether for adults or children. Based on this result, strawberry irrigated with water rich in MCs could pose a serious health risk. In a previous study by Liang et al. (2021), the EDI of MC in rice grains exceeded the TDI (1.148 times) when irrigated with water contaminated with 100 μg L − 1 of MC. Additionally, several studies recorded EDI values ​​that exceeded the TDI by 1.65 to 79.75 times in several edible plants (Cao et al 2018a, b, c; Jia et al 2018; Chia et al 2019). Although the inoculation used in the present study showed considerable ability to reduce the bioaccumulation of MC in strawberries, perhaps they are therefore effective in lowering EDI values ​​to a safe level. It is worth highlighting that the EDI values ​​of MCs in sterile soil were higher than those in non-sterile soil for *Triticum aestivum* and *Vicia faba* (Elmehdi redoune). These results highlight the protective role of the bacteria of certain plants in mitigating the health risk of MCs when consuming products contaminated by MCs.

**Table 2:** Estimated daily intake value for adult and child

	Treatment	0 µg/L	10 µg/L	20 µg/L	CDL µg/L	10 µg/L + *Ensifer sp.*	20 µg/L + *Ensifer* sp.	CDL µg/L + *Ensifer* sp.	10 µg/L + *Shinella* sp.	20 µg/L + *Shinella* sp.	CDL µg/L + *Shinella* sp.	10 µg/L + *Stutzerimonas* sp.	20 µg/L + *Stutzerimonas* sp.	CDL µg/L + *Stutzerimonas* sp.
EDI	Adults	0^a^	1.587 ± 0.133^c^	2.366 ± 0.204^d^	0.849 ± 0.144^b^	0^a^	0^a^	0^a^	0^a^	0^a^	0^a^	0^a^	0^a^	0^a^
	Children	0^a^	1.997 ± 0.278^c^	2.981 ± 0.425^d^	1.119 ± 0.301^b^	0^a^	0^a^	0^a^	0^a^	0^a^	0^a^	0^a^	0^a^	0^a^

Different letters indicate significant differences at *p* < 0.05

### Plant growth and morphology

**Fig. 4:**
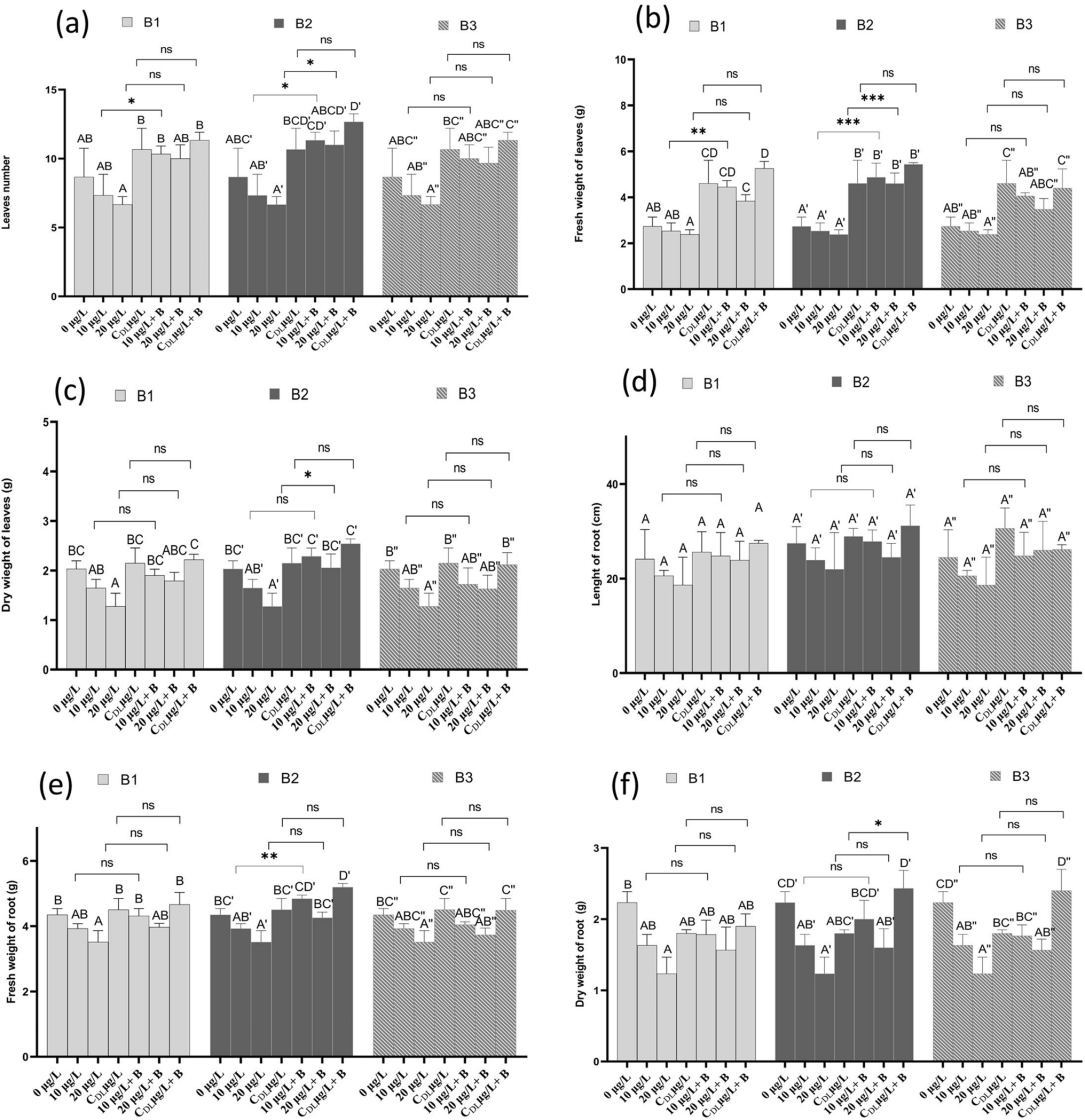
Effects of microcystins in *F. vulgaris* development. Number of leaves per plant (**a**), fresh weight of leaves (**b**), dry weight of leaves (**c**), length of roots (**d**), fresh weight of roots (**e**), and dry weight of roots (**f**) after irrigation with 10 and 20 µg/L of MCs and treatment with bacteria B1, B2 and B3 for 90 days. Values are expressed as mean ± standard error (*n* = 6). Values with the same letters in each column indicate that there is no significant difference (*p* < 0.05) by Tukey's test for each treatment: ns, not significant; ** *p* < 0.01, moderate differences moderate significant differences; *** *p* < 0.001, highly significant differences between microcystin exposure and bacteria in the presence of MC for each treatment group using two-way ANOVA

For the aerial part of strawberry plants, the number of leaves showed a significant increase of approximately 10, 10.33, and 11.36 g after treatment with 10 µg/L + B3, B2, and B1, respectively, and 9.67, 11, and 10 g after treatment with 20 µg/L + B3, B2 and B1, respectively, and 11.34, 12.67 and 11.33 g after treatment with 20 µg/L + B3, B2 and B1, respectively (Fig. 4a). The two-way ANOVA revealed no significant interaction for the number of leaves between the treatments with concentrations of MCs and the same concentrations of MCs in the presence of the bacteria B1, B2, and B3. However, exceptions were observed with treatments at 10 µg/L, 10 µg/L + B1, 20 µg/L, and 20 µg/L + B1, which displayed a weakly significant interaction (Fig. 4a). For the fresh weight of leaves, the results indicated a significant increase of approximately 4.06, 4.45, and 4.87 g following treatment with 10 µg/L + B1, B2, and B3, respectively. Similarly, a rise of 3.48, 3.84, and 4.60 g was observed after treatment with 20 µg/L + B3, B2, and B1, respectively. Additionally, a rise of 4.40, 5.26, and 5.44 g was noted after treatment with 20 µg/L + B3, B2, and B1, respectively. Notably, the interaction was highly significant for the fresh weight of leaves between treatments of 10 and 20 µg/L and between 10 µg/L + B2 and 20 µg/L + B2, respectively (Fig. 4b).

Exposure of plants to 10 µg/L and 20 µg/L along with B1, B2, and B3 bacteria resulted in a notable increase in leaf dry weight (Fig. 4c). However, the interaction did not reach significance for leaf dry weight between treatments with MCs concentrations and the same MCs concentrations in the presence of bacteria B1, B2, and B3, except for the 20 µg/L and 20 µg/L + B1 treatments, which exhibited a marginally significant interaction (Fig. 4c).

**Fig. 5:**
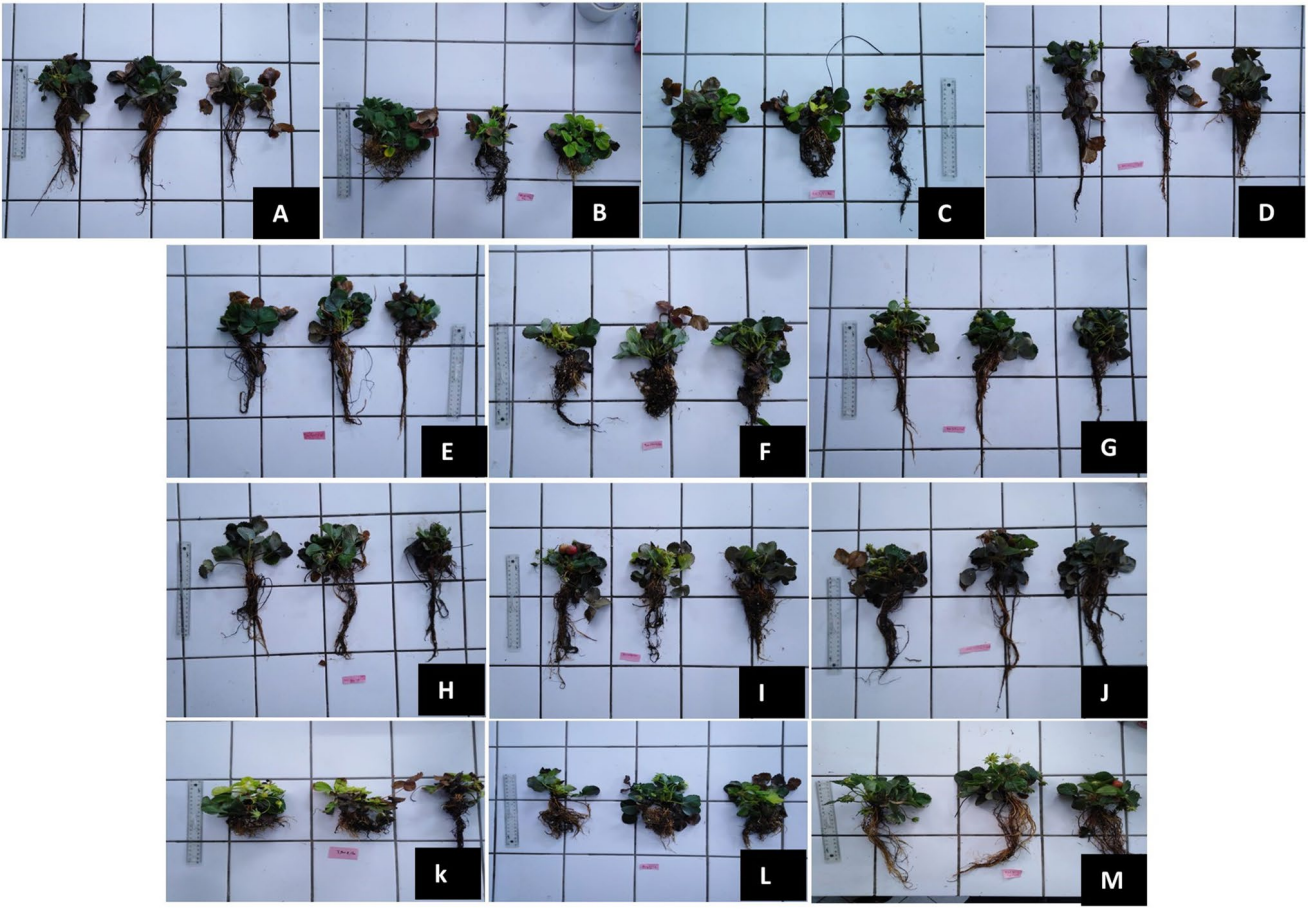
Images of strawberry plants after three months of hydroponic culture. **A** plants were watered with microcystin/bacteria-free water, **B** with 10 µg/L of MC, **C** with 20 µg/L of MC, **D** with CDL µg/L, **E** with 10 µg/L of MC in the presence of B1 bacteria, **F** with 20 µg/L of MC in the presence of B1 bacteria, **G** with CDL µg/L in the presence of B1 bacteria, **H** with 10 µg/L of MC in the presence of bacterium B2, **I** with 20 µg/L of in the presence of bacterium B2, **J** with CDL µg/L in the presence of bacterium B2, **K** with 10 µg/L of MC in the presence of bacterium B3, **L** with 20 µg/L of in the presence of bacterium B3, **M** with CDL µg/L in the presence of bacterium B3

**Fig. 6:**
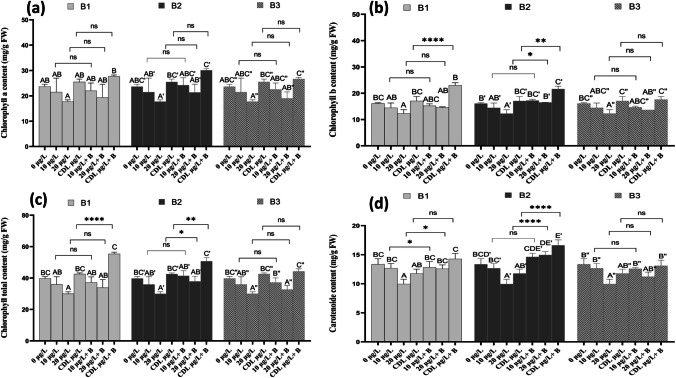
Chlorophyll a (**a**), chlorophyll b (**b**), total chlorophyll (**c**), and carotenoid content (**d**) in *F. vulgaris* leaves exposed to 10 and 20 µg/L MC in MC-contaminated dam water and after treatment with bacterium 1 (B1), bacterium 2 (B2) and bacterium 3 (B3) for 60 days. Bars represent mean values. Error bars represent standard deviations. One-way ANOVA indicates a significant difference (*p* < 0.05) by different letters between control and treatments. The two-factor ANOVA indicates ** *p* < 0.01, or *** *p* < 0.001 a significant difference between exposure times for each treatment group. ns: no significant difference

The results in Figs. 5d, e, f and 6, show that root length, fresh weight, and dry weight of root decreased after exposure to 10 and 20 µg/L MCs and to water from the dam lake contaminated with MCs. Root length decreased non-significantly (p > 0.05), by 24.60 and 25.98 g compared with the control (28.15 g) after exposure to 10 and 20 µg/L of MCs for 90 days, respectively (Fig. 5d). Treatment with the three bacteria (B1, B2, and B3) did not show a significant increase in root length. Bacterium B2 showed the greatest increase in root length (32.54 g) when treated with CDL + B2 µg/L (Fig. 5d). The interaction was not significant for root length between treatment with MCs concentrations and the same MCs concentrations in the presence of MCs biodegrading bacteria B1, B2, and B3 (Fig. 5d). Root fresh weight showed a significant decrease after exposure to 10 and 20 µg/L of MCs compared with the control. Whereas treatment with all three bacteria showed an increase in root fresh weight. This increase was in the order of 4.04, 4.31, and 4.84 g after treatment with 10 µg/L + B3, B1 and B2, respectively. These were 3.73, 3.93, and 4.25 g after treatment with 20 µg/L + B3, B1, and B2, respectively, and 4.49, 4.66, and 5.19 g after treatment with CDL µg/L + B3, B1 and B2, respectively (Fig. 5e). The interaction was not significant for root length between treatment with MCs concentrations and the same MCs concentrations in the presence of MCs biodegrading bacteria B1, B2, and B3 (Fig. 5e). With the exception of 20 µg/L and 20 µg/L + B1, which showed a weakly significant interaction (Fig. 5e). Similarly, for the dry weight of the root, treatment with the three bacteria showed a significant increase in this parameter compared with the control (Fig. 5f). The highest values were recorded after treatment with 10 µg/L + B2 (1.93 g), CDL µg/L + B3 (2.43 g), and CDL µg/L + B2 (2.4 g) (Fig. 5f). The interaction was not significant for root length between treatment with MCs concentrations and the same MCs concentrations in the presence of MCs biodegrading bacteria B1, B2, and B3 (Fig. 5f). With the exception of CDL µg/L and CDL µg/L + B1, which showed a weakly significant interaction (Fig. 5f). The findings of this study unveiled that strawberry plants when exposed to elevated concentrations of MCs for a duration of 90 days, specifically at levels of 10 and 20 g/L, as well as water in dam Lake MC-contaminated, displayed adverse morphological changes. For instance, a recent study focusing on strawberry plants in hydroponic culture revealed that exposure to extracts containing MCs at concentrations of 1, 5, 10, and 20 µg/L for 90 days resulted in negative morphological changes, impacting leaf number, fresh and dry leaf weight, root length, and fresh and dry root weight (Haida et al. 2022). Likewise, Zhou et al. 2018 reported visible alterations, including necrosis, leaf chlorosis, and a significant reduction in root and shoot size, in *Cucumis sativus* after exposure to high concentrations (100 and 1000 µg/L of MCs) for 7 days. These results can be explained by the inhibition of protein phosphatases, because microcystins render inactive the irreversible binding of protein phosphatases, in particular PP1 and PP2A (MacKintosh et al. 1990), these protein phosphatases are well known to be involved in the regulation of several physiological processes by dephosphorylation of regulatory proteins. Inhibition of these proteins in the plant results in leaf malformations, histological changes, and delay in root organ differentiation and vascular cylinder formation with inhibition of lateral primordial root formation (Saqrane et al. 2008). The varied responses of plants to MC may be related to plant species, plant genetics, toxin variant, toxin nature, exposure dose and time, and the microbial and biochemical composition of agricultural soil in which the plant subject is studied (Velikova et al. 2000; Lahrouni et al. 2016; Shen et al. 2019).

The concept of enhancing plant growth through the use of MC-degrading bacteria is a highly promising area of research. This approach presents an economically viable and environmentally safe method to naturally boost crop yields. Numerous studies have explored the influence of microbiota in facilitating plant growth in substrates contaminated with MC. However, it's worth noting that very few, studies have delved into the biodegradation of MCs in substrates while considering changes in concentration and how this relates to improvements in growth, yield, and fruit quality. The results of this study showed that MCs biodegrading bacteria isolated from soil, water, and bloom of cyanobacteria, especially bacteria (B2), can attenuate the effects of MCs on strawberry plants, thereby increasing the growth of plants which results in an increase in the number of leaves, dry and fresh weight of leaves, length of roots, dry and fresh weight of roots. Similarly, Redouane et al. 2021a, b showed that native soil microbes in cultivated areas could protect and enhance the growth of two plant species, *Vicia faba,* and *Triticum aestivum,* after prolonged exposure to high doses of MC. In numerous studies, Plant Growth-Promoting Bacteria (PGPB) have been harnessed to enhance crop yields and shield plants from the detrimental effects of MCs (Asghar et al. 2002; Khalid et al. 2004; Humbert 2009; Zhang et al. 2018; Lobo et al. 2019). These PGPBs contribute to plant growth by facilitating the absorption of essential minerals such as phosphorus and potassium, promoting the synthesis of plant growth hormones like auxin and gibberellic acid (Asghar et al. 2002; Rani et al. 2012), stimulating the production of exopolysaccharides, and enabling atmospheric nitrogen fixation (Rojas-Tapias et al. 2012; Naqqash et al. 2020). The degradation of MCs, based on MCs-degrading bacteria as a control method, can therefore reduce the quantity of MCs reaching the roots and consequently a reduction in the negative effects of these toxins on plants.

### Strawberry pigments content

The results in Fig. 6a, b, c, and d show that photosynthetic pigments are affected by exposure to MCs and water from the dam lake contaminated with MCs. There was a significant decrease (p < 0.05) to 21.48, 17.75, and 25.51 mg/g FW, to 14.47, 12.26, and 17.06 mg/g FW, to 35.95, 30.09, and 42.57 mg/g FW, to 12.66, 9.96 and 11.77 mg/g FW of chlorophyll a, b, total chlorophyll and carotenoids compared with the control (23.63, 16.09, 39.74 and 13.35 mg/g FW), respectively after exposure to 10 and 20 µg/L of MCs for 60 days (Fig. 6a).

Treatment with the three bacteria (B1, B2, and B3) showed a significant increase in the amount of chlorophyll a, b, and total in strawberry leaves. For example, irrigation with dam water contaminated with MCs in the presence of bacteria 1, 2, and 3 showed a significant increase in chlorophyll a to 27.68, 30.12, and 26.66 mg/g FW, respectively compared to the control (Fig. 6a). For carotenoids, treatment with bacteria 2 showed a significant increase in total chlorophyll compared with the control, with the greatest increase observed after treatment with CDL µg/L + B2 of around 16.62 mg/g FW, respectively. As a consequence of the formation of reactive oxygen species (ROS) due to plant stress, photosynthesis becomes the first process to suffer when subjected to stress induced by MCs (Abe et al. 1996). In our research, exposure to concentrations of 10 and 20 µg/L and dam lake water contaminated by MCs led to a reduction in chlorophyll a, b, and total, and carotenoids in the leaves of strawberry plants. This outcome aligns with the findings of Haida et al. (2022), who observed a decrease in photosynthetic pigments in the same plant species (strawberry) following exposure to concentrations of 1, 5, 10, and 20 µg/L in a hydroponic environment. These pigments play indispensable roles in photosynthesis, exerting a direct influence on plant growth and development (Cao et al. 2018c; Xiang et al. 2020). The observed reduction in the concentration of photosynthetic pigments in *F. vulgaris* leaves may be attributed to various factors. It could stem from a malfunction in the Photosystem II (PSII), hindrances in the electron transport chain related to the stimulation of the reaction center, or exposure to excessive light. Factors such as diminished mineral absorption or inhibited reaction center activation may also contribute to this effect (Maxwell and Johnson 2000). The findings of this research demonstrated that the utilization of MC-degrading bacteria showcased their effectiveness in mitigating the adverse impacts of MC. This suggests that in the presence of bacteria, MCs bypass the biodegradation process and no longer become accessible to plants (Redouane et al. 2021a, b). It should be noted that introducing beneficial microflora into plants to alleviate stress improves the synthetic pigment content of the plant, thereby contributing to their resilience under challenging conditions (Kumawat et al. 2023). It is also known that by eliminating MCs by bacteria, this reduces the toxicity and stress induced by these toxins, and therefore strawberry plants can benefit from other potential cyanobacterial bioactive metabolites that could be present in the crude extract. Thus, the biological activity of the crude extract might not be limited to MCs but also to the interaction of other unidentified cyanobacterial bioactive metabolites with the plant. Since only MCs were quantified in the crude extract as the main bioactive compounds, this can increase the photosynthetic performance of the plant by increasing the chlorophyll pigment content. In the light of this work, we can hypothesize that the increase in the content of chlorophyll pigments could indicate a capacity of MCs-degrading bacteria in alleviating stress and consequently improving plants grown in the presence of this type of bacteria. This could be considered a pioneer study, as previous research focused on the degradation of MCs by bacteria with this capability. However, until now, there has been a gap in the literature regarding the application of these bacteria directly to crops as a means to increase MC degradation in the field. Hence, our findings represent a significant step forward in terms of quantifiable progress, offering a biological and cost-effective approach for the removal of MCs while concurrently enhancing plant growth, yield, and fruit quality.

### Strawberry yield parameters

**Fig. 7:**
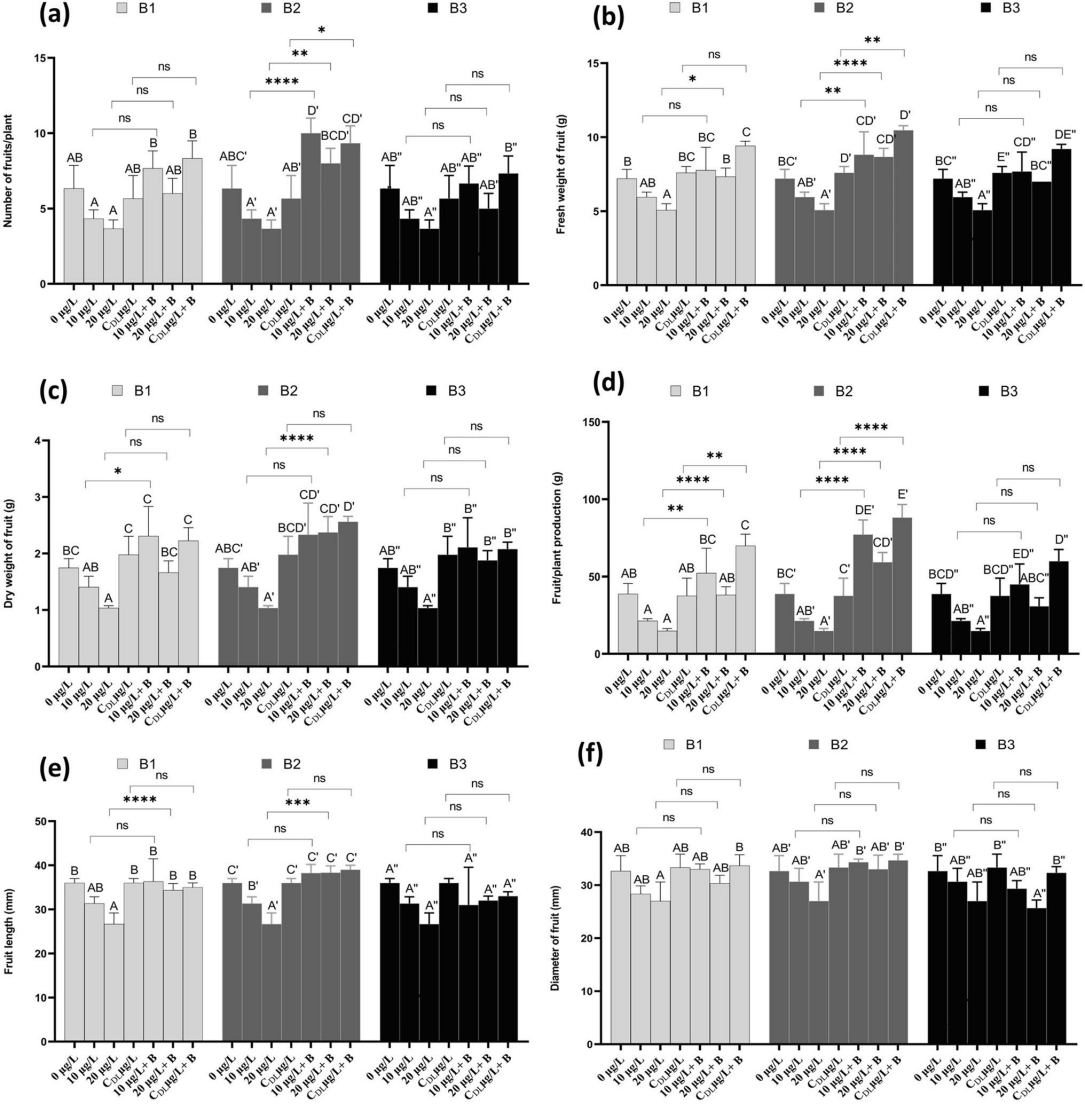
Number of fruits per plant (**a**), fresh weight (**b**), dry weight (**c**), fruit/plant production (**d**), length of fruit (**e**), and diameter of fruit (**f**) in *F. vulgaris* fruits exposed to MC for 60 days and treated with bacterium 1 (B1), bacterium 2 (B2) and bacterium 3 (B3). Bars represent mean values. Error bars represent standard deviations. One-way ANOVA indicates a significant difference (*p* < 0.05) by different letters between the control and treatments. two-way ANOVA indicates ** *p* < 0.01, or *** *p* < 0.001 significant difference between exposure time for each treatment group. ns: no significant difference

**Fig. 8:**
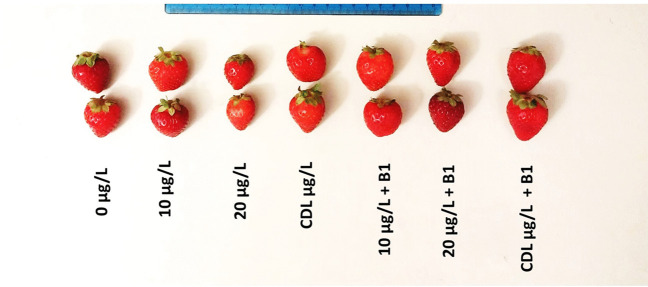
Image of strawberry fruit after plant exposed to three months of hydroponic culture. Fruit plants were watered with microcystin/bacteria, with 10 µg/L of MC, with 20 µg/L of MC, with CDL µg/L in the presence and absence of bacteria

Exposure of strawberry plants to MC (MC) resulted in a significant decrease (*P* < 0.05) in fruit quality and yield parameters compared to the control (Figs. 7a, b, c, d, e, f and 8). The number of fruits per plant showed a significant increase after exposure to dam lake water contaminated by MCs and treatment with the three bacteria B1, B2, and B3 (Fig. 7a). For the fresh weight of fruits, the results showed a significant increase of approximately 7.66, 8.81 and 6.66 g after treatment of plants exposed to 10 µg/L by bacteria B1, B2 and B3, respectively. and 7.37, 8.42, and 7.18 g after treatment of plants exposed to 20 µg/L by bacteria B1, B2, and B3, respectively. The results showed a significant increase in fruit dry weight compared to the control after treatment of plants exposed to MCs with B1, B2, and B3 (Fig. 7c). The results in Fig. 7d show that the fruit produced per plant showed a significant increase in this parameter when treated with MCs in the presence of the three bacteria. The interaction was highly significant for the fruit per plant between treatment with concentrations of MCs and the same concentrations of MCs in the presence of bacteria B1, and B2 (Fig. 7d). In fact, treatment with all three bacteria (B1, B2, and B3) showed a significant increase in fruit length. Bacterium B2 showed the greatest increase in fruit length (39 mm) when treated with CDL + B2 µg/L (Fig. 7e). For the fruit diameter, treatment with the three bacteria showed a significant increase in this parameter compared with the control (Fig. 7f). Our results indicate that extracts from *Microcystis aeruginosa*, with concentrations of 10 and 20 µg/L of MCs, along with dam water contaminated by MCs, exert a detrimental influence on both the quality and yield of strawberry plants. It's important to emphasize that the presence of MCs gives rise to physiological issues that curtail growth and result in reduced yields. This research represents a groundbreaking application of microorganisms for control purposes, underscoring a direct correlation between MC degradation and the improvement of fruit quality. However, our findings showed that the use of MC-degrading bacteria bestows significant advantages on plants, enhancing both fruit yield and quality. This translates into a substantial improvement in plant growth, even in the face of elevated MC concentrations when these bacteria are introduced. In addition to their ability to eliminate MCs, these bacteria may likely exhibit traits characteristic of plant growth-promoting bacteria. Through the secretion of phytohormones, as well as the production of exopolysaccharides and siderophores, which notably accelerate plant growth (Saharan and Nehra 2011). Furthermore, the properties of these bacteria govern the translocation of toxic elements to the plant, reducing the production of ROS and, as a result, mitigating oxidative stress (Kang et al. 2014).

### Nutrient content of strawberry fruit (*Fragaria vulgaris*)

**Fig. 9:**
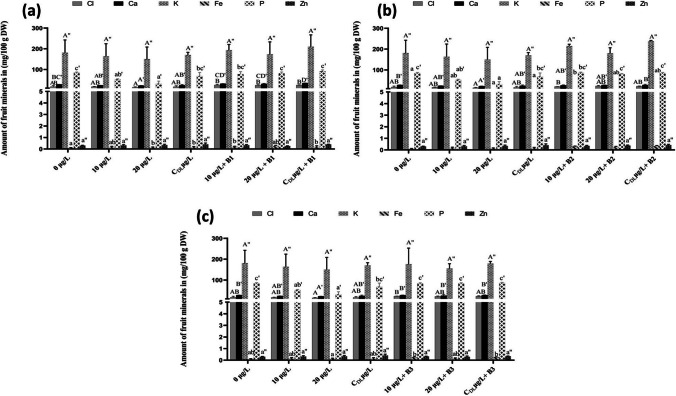
Mineral content in fruits after treatment with bacterium 1 (**a**), after treatment with bacterium 2 (B2) (**b**), and after treatment with bacterium 3 (B3) (**c**) in *F. vulgaris* fruits exposed to MC for 60 days. Bars represent mean values. Error bars represent standard deviations. One-way ANOVA indicates a significant difference (*p* < 0.05) by different letters between the control and treatments

The different concentrations of MCs, as well as water contaminated with MCs, had a negative effect on the content of mineral elements (Cl, Ca, K, Fe, P et Zn) in strawberry plant fruits, as shown in Fig. 9. For fruit from plants treated with bacterium 1 (B1), Ca, Cl, Fe, and P levels showed a significant difference between the different treatments and the control. Among the different treatments, co-inoculation with isolate B1 + dam lake water showed superiority over the other treatments. The highest values were recorded after treatment with CDL µg/L + B1 for Cl (25.14 mg/100 g of DW), Ca (34.92 mg/100 g of DW), K (230.3 mg/100 g DW), Fe (0.20 mg/100 g DW), P (91.5 mg/100 g DW) and Zn (0.41 mg/100 g DW) compared with the control 16.8, 24.39, 203.6, 0.16, 68.73, 0.41 mg/100 g DW, respectively (Fig. 9a). For the fruits of plants treated with bacterium 2 (B2), the levels of Ca, Cl, Fe, and P showed significant differences between the different treatments and the control. Among the different treatments, inoculation with the B2 isolate and water from the reservoir showed superiority over the other treatments. The highest values were recorded after treatment with 20 µg/L + B2 for Cl (25.46 mg/100 g DW), with CDL µg/L + B2 for Ca (28.12 mg/100 g DW), with CDL µg/L + B2 for K (238. 2 mg/100 g DW), with 20 µg/L + B2 for Fe (0.28 mg/100 g DW), with CDL µg/L + B2 for P (84.56 mg/100 g DW) and with CDL µg/L + B2 for Zn (0.42 mg/100 g DW) (Fig. 9a).

The application of bacterium 3 (B3) did not show a significant increase in the amount of minerals in strawberry fruit, except phosphorus content, which showed a significant increase. Minimum P levels of 31.90 mg/100 g DW were found after exposure to 20 µg/L MC. Application of B3 significantly increased the P content of plant fruits from 83.43 and 86.43 mg/100 g DW after exposure to 10 µg/L and CDL respectively. The highest P content of 84.9 mg/100 g DW was recorded under the control treatment (Fig. 9b). Application of Bacterium 3 (B3) to degrade MCs and improve nutrient quality showed a small increase in the amount of minerals in strawberry fruit. Maximum levels of Cl are 22.40 mg/100 g DW, Ca is 28.57 mg/100 g DW, K is 179.45 mg/100 g DW, Fe is 0.27 mg/100 g DW, de is 85.10 mg/100 g DW and Zn is 0.34 mg/100 g DW were found after exposure to CDL µg/L + B3. of MC (Fig. 9c). Consumers make food choices based on sensory attributes, and the nutritional value of vegetables is crucial for human health. Strawberries are renowned for being rich in carbohydrates, proteins, fiber, vitamins, minerals, and phytochemicals (Dahl et al. 2012; Hacisalihoglu et al. 2021). However, our study findings revealed a reduction in the mineral content of the fruit following a 90-day exposure to MCs at concentrations of 10 and 20 µg/L, as well as MC-contaminated dam water. This aligns with the observations made by Dahl et al. 2012, and Liang et al. 2021, who found that high MC concentrations significantly diminish the nutritional content of the fruit, including essential elements such as chlorine (Cl), calcium (Ca), potassium (K), iron (Fe), zinc (Zn), and phosphorus (P). The results obtained in this research align with the findings made by Mugani et al. (2022), where they showed that the presence of rhizospheric bacteria in the soil had a contrasting impact compared to MC on *Pisum sativum* plants. When microcystin enters plants, it can disrupt their metabolism and induce several adverse effects, including a decrease in mineral levels in fruits. This decrease can be attributed to various mechanisms. Firstly, microcystin may interfere with transporters and ion channels involved in nutrient absorption, thereby reducing the uptake of minerals by the plant. Additionally, microcystin can alter the permeability of cell membranes, disrupting the transport of nutrients within the plant. Moreover, microcystins can directly damage plant cells, impairing their ability to absorb and accumulate nutrients necessary for growth and fruit development. Additionally, microcystin-induced disruptions in photosynthesis, a vital process for organic matter and nutrient production in plants, can further reduce the availability of nutrients essential for fruit formation. Inoculating the roots of hydroponically cultivated strawberries with MC-degrading strains (B1, B2, and B3), demonstrated a impact on the nutritional levels of strawberry fruit (*Fragaria vulgaris*) contributing to mitigating the damage caused by MCs on plants. The enhancement in the nutritional quality of strawberry fruit as a result of the MC-degrading bacteria employed in this study can be attributed to the capability of B1 and B2 bacteria to break down MCs, which are known to impact the photosynthesis, growth, and absorption of minerals by the roots.

### Sugar and protein in strawberry fruit (*Fragaria vulgaris*)

**Fig. 10:**
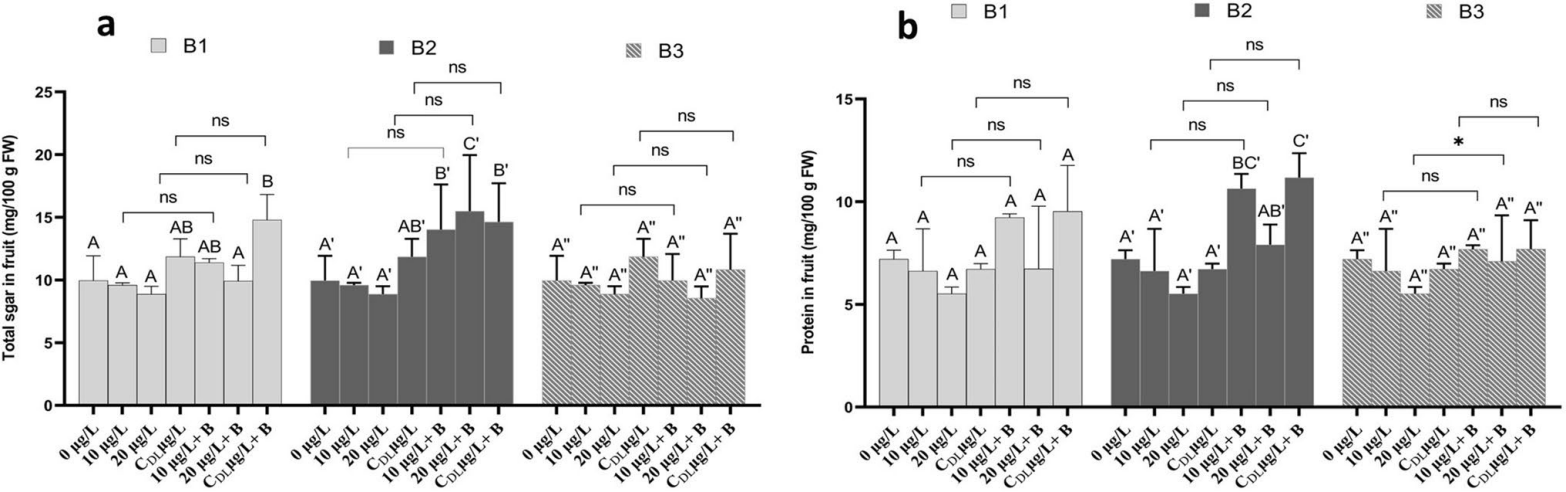
Sugar content (**a**) and Protein content (**b**), in the fruit of *F. vulgaris* exposed to MCs and after treatment with bacterium 1,2 and 3 (B1, B2, and B3) for 60 days. The bars represent the mean values. The error bars represent the standard deviations. One-way ANOVA indicates a significant difference at (*p* < 0.05) between the control and the treatments by different letters (letter x, x’, and x’’). The two-way ANOVA indicates ** *p* < 0.01, or *** *p* < 0.001 significant difference between exposure time for each treatment group. ns: no significant difference

The results showed that the bacterium 1 (B1) and 2 (B2) treatments resulted in a significant improvement (*P* < 0.05) in the biochemical parameters of the strawberry fruit, namely protein, and sugar, compared with the control (Fig. 10). The results of the analysis of total sugars in strawberry fruit showed that irrigation with water containing 10 and 20 µg/L of MC (MC) resulted in a slight decrease in total sugar content in strawberry fruit compared with the control. However, the sugar content in fruit from plants irrigated with MC-contaminated dam lake water was higher than the control. Total sugar content decreased from 9.96 g/100 g FW in fruit from control plants to 8.86 g/100 g FW in fruit from plants irrigated with 20 µg/L of MC (Fig. 10a). Inoculation of the roots with bacteria 1 (B1) and 2 (B2) resulted in a significant improvement (*P* < 0.05) in the sugar content of the strawberry fruit. The greatest improvement was recorded in the fruits of plants treated with 20 µg/L + B2, with 15.48 g/ 100 g FW in sugars, by CDL µg/L + B1, with 14.80 g/ 100 g FW in sugars, and by CDL µg/L + B2 14.63 g/ 100 g FW (Fig. 10a). No significant interaction was recorded between the treatments of the MCs used and the bacteria employed with the same treatments (Fig. 10b). The concentration of Mc in the treatment, the more the protein content decreased (Fig. 10b). The application of bacterium 2 (B2) treatments had a significant effect (*P* < 0.05) effect on the protein content in the fruits of strawberry plants. Maximum protein contents (10.63 and 11.17 g/100 g FW) were measured after treatment with 10 µg/L + B2 and CDL µg/L + B2. No significant interaction was recorded between the treatment with concentrations 10, 20, and CDL µg/L and the treatment with the same concentrations in the presence of the three bacteria (B1, B2, and B3). Except 20 µg/L and 20 µg/L + B3 treatments which showed a weakly significant interaction (Fig. 10b). MCs being water-soluble, pose severe limitations on the use of water in agriculture after a 60-day exposure period, the presence of 10 and 20 µg/l of MCs led to a substantial reduction in the levels of soluble sugar, and protein within strawberry fruits. In a similar work, research investigations have illustrated that MCs, particularly at concentrations of 10, 100, and 1000 µg/l, markedly diminished the content of sugar, and organic acids in fruits like *L*. *esculentum* and cucumbers, as observed in studies conducted by Zhu et al. (2018) and El Khalloufi et al. (2012). When a plant is subjected to stress, such as stress induced by MCs, it can increase its sugar content for several reasons, sugars can serve as protective metabolites by acting as antioxidants or helping to stabilize membrane cells, which helps the plant to better resist stress. As well, sugars are an important source of energy for plants. By increasing their sugar content, plants can have more energy to activate defense or recovery processes. Changes in sugar content can serve as a signal to trigger defense or regulatory responses in the plant, helping it adapt to stress. Numerous research endeavors have provided evidence that when plants are grown in the presence of MCs, MC-tolerant *Rhizobium* strains play a protective role, promoting increased nitrogen uptake. This, in turn, leads to enhanced plant growth, subsequently improving fruit quality and overall production, as highlighted by Lahrouni et al. (2016). The presence of bacteria can reduce the bioaccumulation of MCs and therefore a reduction in oxidative stress and consequently balanced sugar values in fruits. The study marks the first example of elucidating the influence of MC-degrading bacteria on the nutritional quality of strawberries, with a particular focus on sugars and proteins, when these plants are subjected to contamination by MCs.

### Polyphenols and vitamin C in strawberry fruit (*Fragaria vulgaris*)

**Fig. 11:**
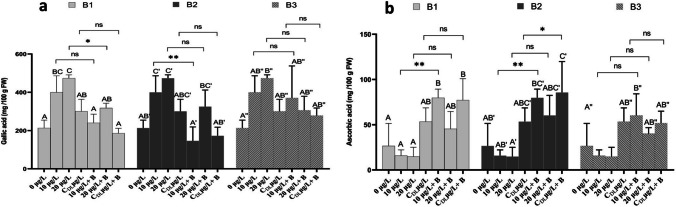
Polyphenols content (**a**) and Vitamin C content (**b**), in the fruit of *F. vulgaris* exposed to MCs and after treatment with bacterium 1,2 and 3 (B1, B2, and B3) for 60 days. The bars represent the mean values. The error bars represent the standard deviations. One-way ANOVA indicates a significant difference at (*p* < 0.05) between the control and the treatments by different letters (letter x, x’, and x’’). The two-way ANOVA indicates ** *p* < 0.01, or *** *p* < 0.001 significant difference between exposure time for each treatment group. ns: no significant difference

Polyphenol levels were systematically higher in fruit from plants irrigated with MCs compared with the control (Fig. 11a). The lowest polyphenol content (473.7 mg/ 100 g FW) was found in the fruits of seedlings treated with 20 µg/L MC, whereas the polyphenol content after exposure to 10 µg/L MC was 399.64 mg/100 g FW in the absence of MC-degrading bacteria. In the presence of bacteria, polyphenol levels decreased significantly. For bacterium 1 (B1), the levels recorded were 240.13, 317.79, and 186.1 mg/ 100 g FW after treatment with 10 µg/L + B1, 20 µg/L + B1 and CDL + B1, respectively. Only the 20 µg/L and 20 µg/L + B1 treatments showed a lower significant interaction (Fig. 11a). Bacteria 2 (B2) showed the greatest increase in polyphenol content, with levels of 146.14, 325, and 172.76 mg/ 100 g FW after treatment with 10 µg/L + B2, 20 µg/L + B2 and CDL + B2, respectively. The interaction was highly significant between the fruits of plants treated with 10 µg/L of MC and 10 µg/L + B2 (Fig. 11a). Treatment of the plants with bacterium 3 (B3) showed a slight increase in polyphenol content. The levels recorded were 370.32, 305.90, and 278.44 after treatment with 10 µg/L + B3, 20 µg/L + B3, and CDL + B3, respectively. The interaction was not significant between the treatments in MCs and the same treatments inoculated with B3 bacteria (Fig. 11a). Exposure of *F. vulgaris* fruits to 10, 20 µg/L MC and to MC-contaminated water modulated the accumulation of total vitamin C content with increasing toxin levels (Fig. 11b). Fruits from plants inoculated with B1, B2, and B3 showed an increase in vitamin C content, with B2 being more efficient at producing vitamin C than B1 and B3. Fruits from plants treated with isolate B2 showed an increase in vitamin C content to 79.78, 60.23, and 85.59 mg of gallic acid/100 g FW after treatment with 10 µg/L + B2, 20 µg/L + B2, and CDL + B2, respectively. Fruit plants treated with isolates B1 and B3 and MCs showed a slight increase in vitamin C content compared with the control. The highest values (79.78 and 60.23 mg of gallic acid/100 g FW) were recorded after treatment with 10 µg/L + B1 and 10 µg/L + B1 of MC, respectively. The interaction was not significant between the treatments of MCs alone and the same treatments inoculated with B3 bacteria. The interaction was significant between 10 µg/L and 10 µg/L + B1 for bacterium 1 (B1) and between 10 µg/L and 10 µg/L + B2 and between CDL and CDL + B2 for bacterium 2 (B2) (Fig. 11b).

MCs can potentially harm various physiological and biochemical processes within strawberry plants Haida et al. (2022). When exposed to adverse environmental conditions, encompassing both abiotic and biotic factors, plants typically react by increasing oxidative processes and elevating the production of ROS (Noctor and Foyer 1998; Codd 2000; Apel and Hirt 2004; Chen et al. 2004; Yin et al. 2005). To safeguard themselves against the detrimental effects of MCs, plants employ antioxidants like ascorbic acid and polyphenols (Hu and Kitts 2005; Prieto et al. 2011; El Khalloufi et al. 2012). Our research findings demonstrate a significant rise in polyphenol levels in strawberry fruits after 90 days of exposure to 10 and 20 µg/l of MC. Likewise, in a study conducted by Drobac et al. (2017) determined that MC leads to the accumulation of polyphenols in *Capsicum annum* fruits, which increases the activity of the antioxidant system of these fruits. In contrast, when strawberry plants were inoculated with MC-degrading bacteria, particularly bacterium 2 (B2), a reduction in polyphenol concentrations was noted in comparison to the sterile control group without MCs. Conversely, an increase in the number of bacterial strains involved led to a decrease in polyphenol concentration in *Vicia faba* and *Pisum sativum*, indicating that the presence of these bacteria mitigated the stress associated with MCs, as noted in studies by El Khalloufi et al. (2013) and Mugani et al. (2022). Additionally, Kang et al. (2014) demonstrated that PGPB exacerbated plant stress conditions by reducing total polyphenol levels when compared to a sterile control. Because they inhibit enzymes and trap trace elements involved in the creation of free radicals, polyphenols are useful in lowering the production of ROS. They also reinforce or protect antioxidant defense pathways and function as ROS scavengers (Drobac et al. 2017; Šamec et al. 2021).

The surplus production of ROS resulting from stress, including superoxide radical anion (O_2_^−^), hydrogen peroxide (H_2_O_2_), hydroxyl radicals (OH^−^), singlet oxygen (^.1^O_2_), and reactive nitrogen species (RNS), can be effectively captured and rendered harmless by vitamin C, as documented in various studies (Liso et al. 1984; Gallie 2013; He et al. 2017). The outcomes of this research indicate that after 90 days of exposure to 10 and 20 µg/l of MCs, followed by the harvesting of strawberries, a notable reduction in the vitamin C content of the strawberries was observed. Likewise, in a study conducted by Machado et al. (2017), a significant reduction in the vitamin C concentration of carrots was observed after exposure to 10 µg/L of MCs. This decline in vitamin C content was attributed to the oxidative effects of MCs, as they are believed to exacerbate induced oxidative stress. However, our research shows that when strawberry plants were introduced to MC-degrading bacterial strains, there was an increase in the levels of vitamin C. This observation aligns with the results from Mugani et al. (2022), where a significant boost in vitamin C content was noted in *Pisum sativum* fruits that were treated with bacterial strains when compared to the control. In plants, ascorbic acid is known as the greatest primary antioxidant. It acts as a primary substrate in the enzymatic pathway of detoxification of ROS. It is also a powerful secondary antioxidant, reducing the oxidized form of α-tocopherol. It has been reported that ROS produced by various types of stress leads to a decrease in ascorbic acid through its oxidation and formation of dehydroascorbic acid (oxidized form) (Noctor and Foyer 1998). The action of bacteria in degrading MC, a process that induces stress in the plant, subsequently elevating the vitamin C concentration. Considering the multifaceted role of vitamin C in plant metabolism and physiology, we can hypothesize that in response to MC-induced oxidative stress, MC-degrading bacteria trigger an upsurge in vitamin C to counteract the damage inflicted by ROS on the plant and bolster vitamin C as a nutritional reserve within the fruit.

## Conclusion

In recent years, hydroponic cultivation of red berries has expanded in Morocco. The majority of these crops are irrigated with water from dam lakes and water storage basins contaminated by MCs. Research into methods of combating the proliferation of cyanobacteria or the degradation of MCs released by these cyanobacteria has developed very rapidly throughout the world. However, from the data synthesized and presented above, it is clear that bacteria with the ability to degrade MCs can be used as a simple and inexpensive biological method. The bacterial treatment resulted in a significant increase in the growth, yield, biochemical constituents, and physicochemical quality attributes of strawberry fruits. However, several gaps in scientific knowledge and technological challenges still limit the widespread application of MC-degrading bacteria and their integration into agricultural practices. The worldwide trend towards the application of MC-degrading bacteria as bioremediators in water without direct application in the substrates used in agriculture has so far focused on a few taxa, often isolated from aquatic environments. Recently, it has become clear that there is a need to investigate other strains of interest for degrading MCs and to develop new innovative and effective formulations that increase crop yields and minimize the environmental impact of MCs. Consequently, further research is needed to evaluate the biostimulant effects of various MCs-degrading bacteria that can also provide a range of bioactive compounds and signaling molecules, in addition to inorganic and organic nutrients. In addition, the search for new taxa of MC-degrading bacteria from soil, blooms, and MC-contaminated waters could provide an original biological alternative. It is therefore important to further broaden the scope for exploring new strains and optimizing bacteria-based techniques.

## Supplementary Information

Below is the link to the electronic supplementary material.

Supplementary file1 (XLSM 31 KB)
